# Active nuclear transcriptome analysis reveals inflammasome-dependent mechanism for early neutrophil response to *Mycobacterium marinum*

**DOI:** 10.1038/s41598-017-06099-x

**Published:** 2017-07-26

**Authors:** Amy Kenyon, Daria Gavriouchkina, Jernej Zorman, Giorgio Napolitani, Vincenzo Cerundolo, Tatjana Sauka-Spengler

**Affiliations:** 1 University of Oxford, Weatherall Institute of Molecular Medicine, Radcliffe Department of Medicine, Oxford, OX3 9DS United Kingdom; 2 University of Oxford, Weatherall Institute of Molecular Medicine, MRC Human Immunology Unit, Radcliffe Department of Medicine, Oxford, OX3 9DS United Kingdom

## Abstract

The mechanisms governing neutrophil response to *Mycobacterium tuberculosis* remain poorly understood. In this study we utilise biotagging, a novel genome-wide profiling approach based on cell type-specific *in vivo* biotinylation in zebrafish to analyse the initial response of neutrophils to *Mycobacterium marinum*, a close genetic relative of *M*. *tuberculosis* used to model tuberculosis. Differential expression analysis following nuclear RNA-seq of neutrophil active transcriptomes reveals a significant upregulation in both damage-sensing and effector components of the inflammasome, including *caspase b*, *NLRC3* ortholog (*wu: fb15h11*) and *il1β*. Crispr/Cas9-mediated knockout of *caspase b*, which acts by proteolytic processing of il1β, results in increased bacterial burden and less infiltration of macrophages to sites of mycobacterial infection, thus impairing granuloma development. We also show that a number of immediate early response genes (IEGs) are responsible for orchestrating the initial neutrophil response to mycobacterial infection. Further perturbation of the IEGs exposes egr3 as a key transcriptional regulator controlling *il1β* transcription.

## Introduction

In 2015 10.4 million people developed tuberculosis and 1.8 million people died from the disease^1^. Although an appropriate immune response generally follows infection, the initial responses often fail to clear the bacteria. In *Mycobacterium tuberculosis* (MTB), the innate immune phase is usually initiated by the phagocytosis of bacteria in the lung by macrophages, followed by the accumulation of macrophages, neutrophils, dendritic cells and innate lymphoid cells^2^. As they are recruited, some cells get infected by the expanding population of mycobacterium and early granulomas start to form^2^. The granuloma is the hallmark lesion of tuberculosis, an organised structure of aggregated immune cells, in which bacteria may persist^3, 4^. By defining the contribution of specific cells in the innate immune response, we are poised to provide a clearer understanding of correlates of innate immunity that may lead to asymptomatic clearance rather than progression to latent and active disease.

The role of neutrophils in mycobacterial infection has been poorly investigated, and their relevance in the pathogenesis remains controversial. In pulmonary tuberculosis, neutrophils are present both in newly forming and established granulomas^5^, but *ex vivo* studies on these cells are hindered by their short half-life, their vulnerability to cryopreservation and the difficulty to isolate them whilst still preserving their activation state^6^. The studies on neutrophils in mammalian models of MTB present conflicting results with some authors suggesting that neutrophils play a protective role in early infection, whilst others report no such effect^6^. Some studies have focused on pathological roles of neutrophils, where excessive neutrophil accumulation is seen in advanced infection and a higher neutrophil count is associated with poorer prognosis^7^.

Zebrafish are naturally susceptible to *Mycobacterium marinum*, the closest genetic relative to MTB^8^. *M*. *marinum* infection in zebrafish has become an accepted model to study host-pathogen interactions in MTB because *M*. *marinum* infection in zebrafish shares the main pathological and histological features as MTB^9^. Zebrafish infection results in the development of necrotic, caseating granulomas, similar to those seen in human MTB infections^4^. In particular, the zebrafish embryo provides an ideal model system to study the innate immune response to mycobacterial infection and potential effectors of asymptomatic clearance, since the adaptive arm develops 2–3 weeks post fertilization^10^.

To identify neutrophil-specific effector mechanisms capable of controlling mycobacterial infection, we have developed a novel transgenic zebrafish model system based on the method developed by Trinh and Chong *et al*., adapted for the *in vivo* biotinylation and the subsequent isolation of the neutrophil nuclei^11^. This model circumvents previous problems with the variability of short-lived neutrophils following isolation from blood by rapidly sequestering the nuclei. Using this approach it was possible to perform genome-wide analysis of the active transcriptome of neutrophils at the onset of *M*. *marinum* infection. We show for the first time that neutrophils fight mycobacterial infection by an inflammasome-dependent mechanism during the initiation of infection. We demonstrate that knockout of *caspase b*, which enhances processing and secretion of il1β, increases bacterial burden and prevents macrophage migration to sites of infection in a process that results in extracellular growth of the mycobacteria. Finally, we show that egr3, an early growth response transcription factor, plays a role in the modulation of transcription of *il1β*.

## Results

### Binary transgenic zebrafish model for genome-wide analysis of neutrophil active transcriptomes

**Figure 1:**
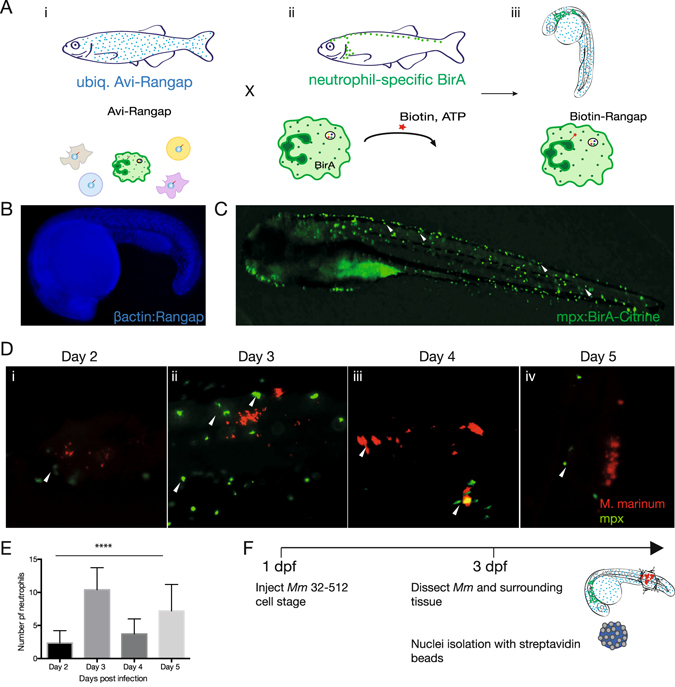
Binary transgenic zebrafish model for regulatory profiling of neutrophils in response to *M*. *marinum*. (**A**) Driver line expressing BirA in neutrophils (i) is crossed with effector line ubiquitously expressing Avi-tagged Rangap (ii) for biotinylation of nuclei. In double transgenic fish, only the nuclei of neutrophils will be biotinylated (iii). (**B**) Effector line with ubiquitously expressed Avi-Cerulean-Rangap. (**C**) Neutrophil-specific BirA driver line with Citrine reporter. (**D**) Time course analysis of neutrophil infiltration of *M*. *marinum*. Embryos were injected with *M*. *marinum* at the 32-512-cell stage, fixed and immunofluorescence carried out for mpx protein at 2 dpi (i), 3 dpi (ii), 4 dpi (iii) and 5 dpi (iv). Neutrophil localisation is indicated by white arrows. (**E**) Neutrophil numbers in proximity to *M*. marinum were quantified at 2–5 dpi. Statistical significance was determined by one way ANOVA (n = 14–18, ****p < *0*.*0001*). (**F**) Infection model for subsequent experiments.

To isolate neutrophil nuclei, we adapted the biotagging method developed by Trinh and Chong *et al*., where target proteins are specifically biotinylated *in vivo* and as a result, the molecular and cellular components associated with them can be isolated using streptavidin beads^11^. In this approach a binary system of transgenic fish is used (Fig. 1A). The biotagging effector line (*Tg*(*βactin:Avi-Cerulean-RanGap*)^*ct700a*^) features ubiquitous expression of an Avi-tagged version of the carboxy-terminal domain of Ran-GTPase-activation protein 1 (Rangap1), fused to a cerulean reporter, which targets the outer surface of the nuclear envelope, Fig. 1Ai,B). In our system, the effector line is then crossed with a biotagging driver line (*TgBAC*(*mpx:BirA*-*2A*-*Citrine*)^*ox121*^) harbouring the gene for *E*. *coli* biotin ligase, BirA, under the regulatory control of a neutrophil-specific myeloperoxidase gene (*mpx*) (Fig. 1Aii,C). In the offspring embryos carrying both biotagging alleles, this approach results in the deposition of biotin to the Avi-tagged Rangap1 and hence the biotinylation of the neutrophil nuclei specifically (Fig. 1Aiii).

The neutrophil BirA transgenic driver line was generated by tol2-mediated BAC transgenesis (see Supplementary Fig. S1), whereas the previously generated ubiquitous effector line uses the zebrafish *β-actin* promoter to drive the expression of Avi-Cerulean-Rangap.

### Neutrophil interaction with *M*. *marinum* increases at 3 days post fertilization

To ensure optimal infection experiments, *M*. *marinum* was transformed with a pmsp12 vector carrying a tdTomato fluorescent reporter and prepared in accordance with previously established methods^8^. Variation in the amount of bacteria in the circulation was minimized by injecting bacteria into the developing cell mass (32–512 cell stage).

To gain a better understanding of the early phase of interaction between neutrophils and mycobacteria, we performed a detailed kinetic study of neutrophil dynamics early in infection. Neutrophil numbers in proximity to the mycobacteria were quantified between day 2 and day 5 post infection and statistical significance of the results assessed using one-way ANOVA test.

We observed that neutrophils started to approach the mycobacteria at 2 days post infection (dpi) in concomitance with their developmental maturation (Fig. 1Di,E). The frequency of neutrophils in proximity of mycobacteria peaked at 3 dpi (****p < *0*.*0001*), although no phagocytosis of the mycobacteria was evident (Fig. 1Dii,E). This is in line with previous studies which have found that neutrophil phagocytosis of *M*. *marinum* is initiated only once granuloma formation has occurred^5^. By 4 and 5 dpi, the number of neutrophils in close proximity to the mycobacteria was reduced (Fig. 1Diii,iv,E). We have therefore chosen to focus our analysis to 3 dpi, given our interest in the earliest role of neutrophils in the containment of infection, even before granuloma formation has occurred.

### Early transcriptional signature of neutrophils as they respond to *M*. *marinum*

To characterise the functional response of neutrophils to *M*. *marinum* infection we performed nuclear transcriptome analysis of neutrophils at 3 dpi. Double transgenic *Tg*(*mpx:BirA-2A-Citrine;* β*actin:Avi-Cerulean-RanGap*)^*ox128*^ fish were in-crossed and embryos were injected with *M*. *marinum* directly into the cell mass (32–512 cell stage). To reveal only the transcriptome of neutrophils responding to the mycobacterium and eliminate the contribution of developing neutrophils found within the proximity of the yolk sac and the intermediate cell mass, aggregates of mycobacterium and the tissue in close proximity to the *M*. *marinum* infection sites were dissected from the tails of 3 dpi embryos (Fig. 1F). Biotagged neutrophil nuclei were isolated using nuclei pulldown^11^, whereby embryos were lysed in a hypotonic buffer, nuclei isolated with streptavidin dynabeads and processed for RNA extraction. We prepared sequencing libraries from polyA-ed nuclear transcripts and subjected them to Next Generation Sequencing. Scatterplots of technical replicates show reproducibility between the 3 independent experiments (see Supplementary Fig. S2).

**Figure 2:**
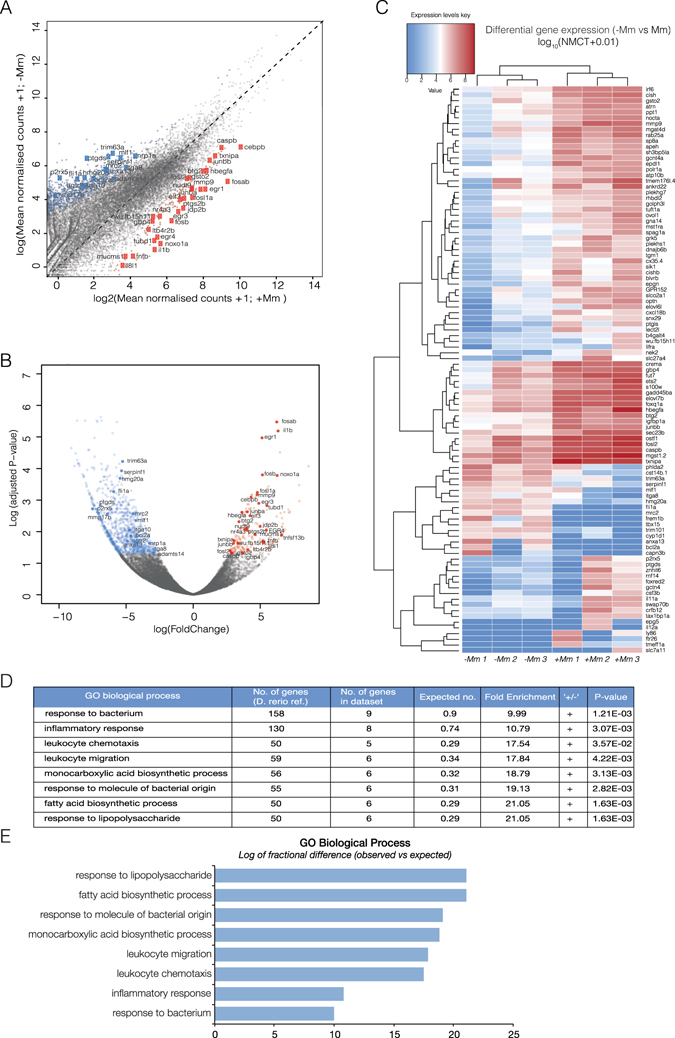
*In vivo* biotinylation of neutrophil nuclei and RNA-seq analysis reveal transcriptomic signature in response to *M*. *marinum* infection. (**A**) Scatterplot of log (Mean DESEq2 normalised counts (NMCT) +1) comparing *M*. *marinum* infected and uninfected controls show 189 upregulated genes (red) and 1100 downregulated genes (blue). (**B**) Volcano plot of DESEq2-generated differential expression analysis of embryos injected with *M*. *marinum* vs uninjected controls shows the relationship between p-value and log fold change (red-upregulated; blue-downregulated; grey-not significantly differentially expressed). (**C**) Heatmap shows the log_10_ (normalised counts (NMCT) +0.01) of selected differentially expressed transcripts (adjusted p-value < 0.05). Red - high expression. White - medium expression Blue - low expression. (**D**) Gene ontology enrichment analysis of upregulated genes following *Mm* infection. (**E**) Bar plot shows significantly overexpressed biological processes.

Differential expression analysis comparing active transcription in neutrophil nuclei of *M. marinum*-infected vs uninfected embryos identified 189 upregulated genes and 1100 downregulated genes with a statistical significance cut-off of *p < *0*.*05* (Fig. 2A–C, see Supplementary Data).

Statistical overrepresentation analysis of enriched genes revealed high significance in association with gene ontology (GO) terms for biological function, clearly reflecting a molecular signature of leukocytes responding to microbial infection (response to bacterium, inflammatory response, leukocyte chemotaxis, leukocyte migration, monocarboxylic acid biosynthesis process, response to molecule of bacterial origin, fatty acid biosynthesis process and response to lipopolysaccharide). This result highlighted the validity of the biotagging approach to reveal dynamic changes in neutrophil transcriptional profiles (Fig. 2D).

### *In situ* hybridisation of selected upregulated genes confirms RNA-seq data

**Figure 3:**
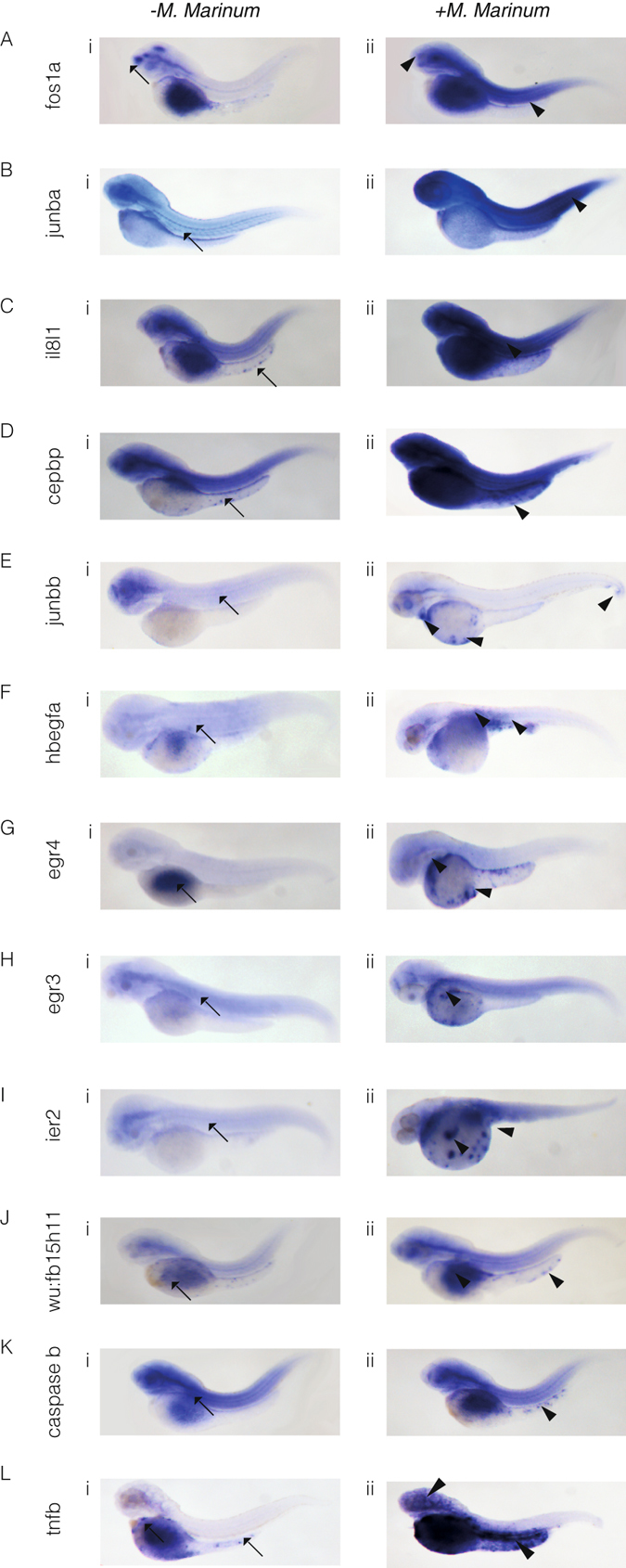
*In situ* hybridisation reveals spatial analysis of *M*. *marinum*-induced upregulated genes. Embryos were injected at the 32-512-cell stage with *M*. *marinum*. Expression patterns and gene transcript levels were analysed by *in situ* hybridisation using digoxigenin-labelled RNA probes, in uninjected controls (**A**–**Li**) and *M*. *marinum*-injected embryos (**A**–**Lii**). Control expression patterns are indicated by the black arrows with black arrowheads used to indicate altered expression patterns for *fos1a* (**A**), *junba* (**B**), *il81a* (**C**), *cepbp* (**D**), *junbb* (**E**), *hbegfa* (**F**), *egr4* (**G**), *egr3* (**H**), *ier2* (**I**), *wu:fb15h11* (**J**), *caspase b* (**K**) and *tnfb* (**L**).

To validate the results of the differential expression analysis on nuclear RNA-seq in *M. marinum* infection and control conditions and gain insight into the expression patterns of some of the upregulated genes, we carried out whole mount *in situ* hybridisation. Images are representative of 2–3 independent experiments, where for each experiment at least 10 embryos were used per *in situ* probe (Fig. 3). Mycobacterial infection led to a systemic increase in the expression of *fosla*, *junba*, *il8l1* and *cepbp* (Fig. 3A–D). *junbb* and *hbegf* upregulation was localised to sites of neutrophil development and areas of bacterial infection (Fig. 3E,F). An increase in *egr4* and *egr*3 expression was seen in the yolk sac, presumably where some *M*. *marinum* has localised, and in particular in the anterior yolk sac, a site of neutrophil production, suggesting the transcriptional regulation of the *egrs* may occur even before the neutrophils arrive in proximity of *M*. *marinum* infection (Fig. 3G,H). *Ier2* shows a similar pattern to the *egrs*, but with punctate expression in the yolk sac and the embryo once again indicating localised expression to *M*. *marinum* infection (Fig. 3I). Both *wu:fb15h11* and *caspase b* transcription appears to increase in the yolk sac and in distinct cells, presumably of myeloid identity (Fig. 3J,K). Finally, an increase in *tnfb* can be seen in the yolk sac and distinct areas of the embryo proper, indicating a localised increase in proximity of *M*. *marinum* infection (Fig. 3L).

### Neutrophils mediate M. marinum killing by inflammasome-dependent mechanism

Further analysis of genes enriched in neutrophil active transcriptomes upon encounter with *M*. *marinum* revealed an upregulation of integral members of the inflammasome complex and additional interacting partners. The inflammasome is a Pattern Recognition Receptor (PRR)-containing complex that acts as the platform for the activation of pro-inflammatory caspase-1, the active form that proteolitically cleaves pro-IL1β and pro-IL18 to generate mature cytokines which are subsequently released from the cell to mediate downstream inflammatory effects^12^. In mammals, the simplest form of the inflammasome includes a NOD-like receptor (NLR), Caspase 1 and if required, the adaptor apoptosis-associated speck-like protein containing a caspase recruitment domain (ASC). NLRs are characterised by N-terminal protein-interaction region that either contains a caspase recruitment domain (CARD; including NOD1, NOD2 and the NLRC3 family) or a pyrin domain (NLRP3 family), a central oligomerisation domain (NACHT) and a C-terminal leucine rich repeat (LRR) domain^12, 13^. Upon activation, the NLRs associate with the adaptor protein (ASC), which also has a caspase recruitment domain (CARD) and a pyrin domain. The NLR complex oligomerises and binds Caspase 1 either in the absence or presence of ASC. Catalytically active Caspase 1 then cleaves IL1β to the mature 17 kDa form.

**Figure 4:**
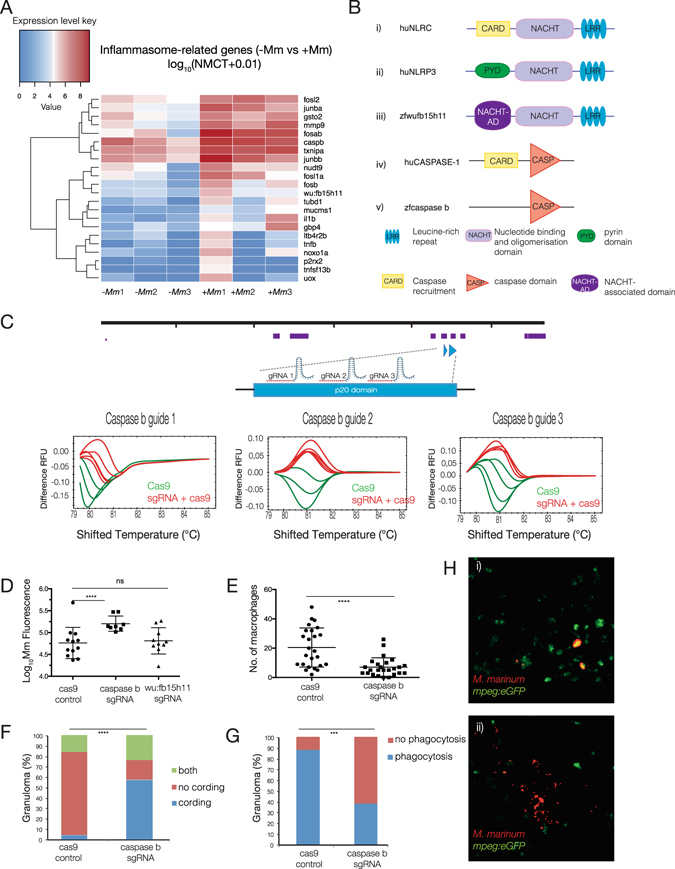
Neutrophils mediate *M*. *marinum* killing by inflammasome-dependent mechanism. (**A**) Heatmap shows the log_10_ (normalised counts (NMCT) +0.01) of selected differentially expressed transcripts of known inflammasome components and related genes (adjusted p-value < 0.05). Red - high expression. White - medium expression. Blue - low expression. (**B**) Schematic representation of the domain organisation of huNLRP3 (i), huNLRC (ii), zfwu:fb15h11 (iii), huCaspase 1 (iv) and zfcaspase b (v). (**C**) *caspase b* sgRNA tests for efficiency in inducing DNA double-strand breaks. sgRNAs were injected into the 1-cell stage and genomic DNA extracted at 24 hpf. High resolution melting curves from 3–4 individual embryos injected either with Cas9 mRNA only (green) or co-injected with Cas9 mRNA and *caspase b* sgRNA (red) are shown. (**D**–**H**) For quantitative analysis of inflammasome components, embryos were injected at the one-cell stage with Cas9 mRNA or sgRNAs to *caspase b* or *wu:fb15h11* followed by injection of *M*. *marinum* at the 32–512 cell stage. (**D**) Bacterial burden of larvae infected at 3 dpi as measured by *Mm* fluorescence intensity (red). Statistical significance was determined by two-tailed unpaired students t-test with Welch’s correction. For *caspase b* sgRNA, ****p < *0*.*0001*. (**E**) Enumeration of macrophage infiltrating *M*. *marinum* at 5 dpi. Statistical significance was determined by two-tailed unpaired students t-test with Welch’s correction (****p < *0*.*0001*). (**F**) Percentage of larvae with cording phenotype at 5 dpi. Statistical significance was determined by chi-squared test (****p < *0*.*0001*). (**G**) percentage of larvae where macrophages phagocytise *M*. *marinum* at 5 dpi. Statistical significance was determined by Fishers exact test (***p < *0*.*001*). (**H**) Representative maximum intensity projections of larvae with cording bacteria with decreased phagocytosis in caspase b knockout embryos (i) vs non-cording controls (ii).

As shown in the heatmap in Fig. 4A, infection lead to an upregulation in core components of the inflammasome including the putative *NLRC3* ortholog (*wu:fb15h11*), *caspase b* and *il1β*, as well as transcription factors previously shown to regulate inflammasome components and other known mediators of inflammasome activation or downstream effectors (Fig. 4A).

A phylogenetic study of NLRs in zebrafish revealed 3 distinct families; NLR-A, orthologous to mammalian NOD-like receptors, NLR-B, orthologous to mammalian NLRP receptors and NLR-C, a group unique to teleost fish^14^. Various NLR orthologs have been identified in fish. The only zebrafish ortholog of NLRP3 identified does not have an N-terminal effector domain so its identification as a true ortholog of human NLRP3 warrants further investigation (Fig. 4Bi). Instead a number of human NLRC3 orthologs were identified including *wu:fb15h11* (Fig. 4Bii,iii). It has previously been shown that two distinct zebrafish inflammatory human caspase 1 orthologs are capable of IL-1β processing, caspase a and caspase b (Fig. 4Biv,v)^15^.

To address the importance of inflammasome activation on bacterial burden, we used genome engineering to knock out several inflammasome candidate genes. Given that most of the genes perturbed were not essential for early development, we devised a highly efficient method whereby multiple (5–6) synthetic guide RNAs (sgRNAs) were designed to target regions encoding domains essential for protein function of a given candidate. We first tested the efficiency of individual sgRNAs by co-injection with Cas9 mRNA into a developing zebrafish embryo at the single cell stage. We used High Resolution Melt Analysis (HRMA) to assay for the mismatches generated in a single embryo by double-stranded DNA breaks and non-homologoues end joining (NHEJ)^16^. Three best performing sgRNAs (75–100% efficiency defined as percentage of embryos assayed indicating mutational event) were used to target the regions encoding p20 and NACHT domains of *caspase b* (Fig. 4C) and *wu:fb15h11* (see Supplementary Fig. S3) genes respectively. Following injections of selected pooled sgRNAs at the single cell stage, embryos were allowed to develop to 32-cell stage and subsequently injected with *M*. *marinum*. Cas9-only injected embryos were used as controls. *M*. *marinum*-infected embryos with or without inflammasome components were then allowed to develop to 3 dpi and *M*. *marinum* fluorescence of individual infection foci (pre-forming granulomas) was quantified as a measure of bacterial burden. Importantly, we found that targeted knockout of *caspase b*, but not *wu:fb15h11* led to a statistically significant increase in bacterial burden (****p < *0*.*0001*) when compared to Cas9-only injected sibling controls, as measured by *M*. *marinum* fluorescence intensity (Fig. 4D). The lack of phenotype in *wu:fb15h11*-deficient zebrafish may be due to a compensatory mechanism, where inflammasome activation would be mediated by any of several other NLR orthologs present in this organism.

To gain further insight into the role of neutrophil-derived il1β on granuloma formation, we investigated whether targeted knockout of *caspase b*, which should result in a reduction of processed il1β, would result in impaired macrophage recruitment to sites of infection. Enumeration of macrophages following caspase b knockout showed a statistically significant reduction in macrophage numbers at 5 days post infection (***p < *0*.*001*) (dpi) (Fig. 4E). Interestingly, there was no change in the frequency of neutrophils from 2 to 5 dpi in the presence or absence of caspase b, suggesting that inflammasome activation is required for the sustained immune response needed for macrophage accumulation, but not for the early recruitment of neutrophils (see Supplementary Fig. S4). Furthermore, in the *caspase b* knockout embryos, *M*. *marinum* exhibited a cording appearance, a feature of extracellular bacteria. We quantified this effect and found a statistically significant increase (****p < *0*.*0001*) in cording mycobacteria in *caspase b* knockout embryos (Fig. 4F). Since cording bacteria are usually associated with a loss of phagocytosis by macrophages^17^ we went on to determine whether knockout of *caspase b* would result in a reduction in phagocytosis of *M*. *marinum*. Images were acquired by z-stack confocal imaging, assembled and analysed for the precise co-localisation of macrophages (green) and *M*. *marinum* fluorescence (red). Quantification of the absence or presence of phagocytosis in infection foci revealed a statistically significant reduction in phagocytosis in *caspase b* knockout embryos (***p < *0*.*001*) (Fig. 4G–H).

### Immediate-early growth response genes regulate early inflammatory processes during *M*. *marinum* infection

**Figure 5:**
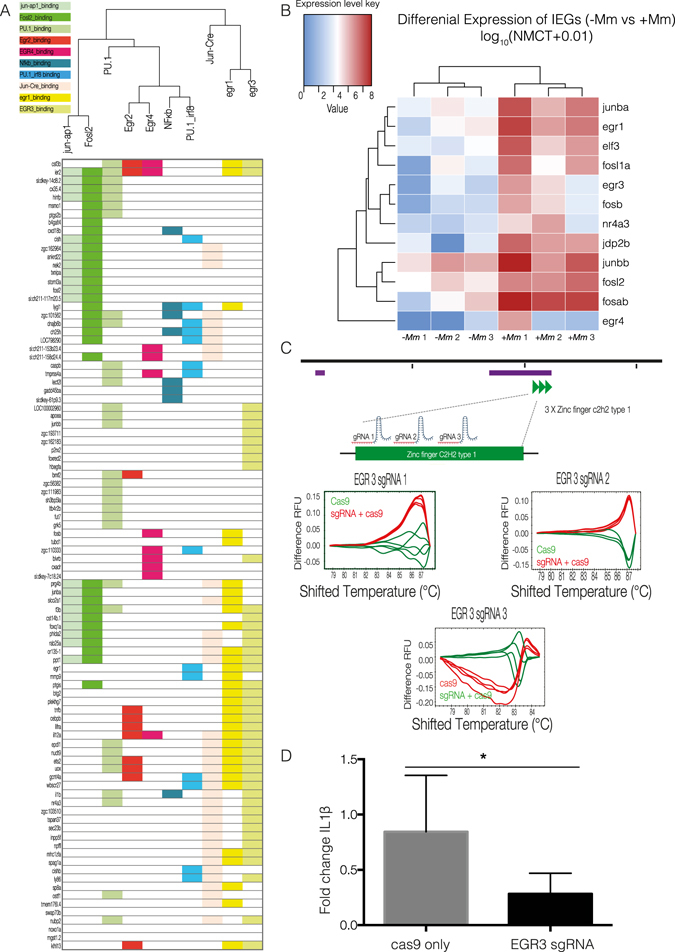
Analysis of transcription factors orchestrating neutrophil response to *M*. *marinum*. (**A**) Transcription factor motif search was performed on upregulated genes by *de novo* analysis using Homer. Dendrogram shows clustering of statistically significant transcription factors and upregulated genes with corresponding transcription factor binding sites highlighted below. (**B**) Heatmap shows the log_10_ (normalised counts (NMCT) +0.01) of selected differentially expressed transcripts for initial early response genes (IEGs) (adjusted *p-value* < *0*.*05*). Red - high expression. White - medium expression Blue - low expression. (**C**) *egr3* sgRNA tests for efficiency in inducing DNA double-strand breaks. sgRNAs were injected into the 1-cell stage and genomic DNA extracted at 24 hpf. High resolution melting curves from 3–4 individual embryos injected either with Cas9 mRNA only (green) or co-injected with Cas9 mRNA and *egr3* sgRNA (red) are shown. (**D**) Bar graph representing il1β mRNA levels measured at 3 dpi by qPCR. Wildtype fish were co-injected with Cas9 mRNA and 3 sgRNAs for targeted knockout of *egr3* or Cas9 mRNA only at the one cell stage, followed by injection of *M*. *marinum* at the 32–512 cell stage. Statistical significance was determined by two-tailed unpaired students t-test with Welch’s correction (*p < *0*.*05*).

Transcription factors and the transcriptional programmes they mediate integrate the molecular events and signalling pathways controlling the state of a specific cell type in response to a pathophysiological insult such as *M*. *marinum* infection. To gain an understanding of the transcription factors governing these networks in the context of neutrophils activation, Homer motif analysis was performed to identify common motifs present in the promoters of genes upregulated in response to *M*. *marinum*. Promoters were defined as regions spanning −500 bp to +1000 bp from the transcription start site (TSS)^18^. This analysis has allowed us to identify statistically overrepresented transcription factor binding sites. Amongst those, the most prominent binding motifs identified were those for EGR and AP-1 family members, as well as for NF-κB. We show that a number of upregulated genes from the RNA-seq data set have promoters with transcription factor binding sites corresponding to the clustered transcription factors in the dendrogram above (Fig. 5A).

A number of the transcription factors identified independently by Homer motif analysis were enriched in the RNA-seq data set^19^. Immediate early response genes (IEGs) encode transcription factors which may be transcribed as a result of early inflammatory immune processes and subsequently regulate the transcription of a wide range of downstream effector genes. Our data show that a number of IEGs respond quickly to regulatory signals coordinating the first line of defence in neutrophils against *M*. *marinum*. In addition to early growth response (egr) family members (*egr1*, *egr3* and *egr4*), we show upregulation of members of the activator protein 1 (AP-1) complex, which is composed of heterodimers of Fos and Jun family members (*fosb*, *junba*, and *junbb*) as well as other Fos family members (*fosla*, *fosl2*, *fosab*) (Fig. 5B).

Based on the Homer network analysis, we hypothesised that some of the transcription factors significantly upregulated in the nuclear RNA-seq dataset may play a role in regulating the expression of *il1β*. We chose to focus on egr3, which has binding sites upstream of the promoter of *il1β* and, importantly, appeared to have an expression pattern that was largely limited to the myeloid component, as shown in the *in situ* hybridisation analysis (Fig. 3H). Egr3 is a member of the EGR family of zinc finger transcription factors that have been implicated in the modulation of the immune response^20^. Three sgRNAs were designed to target the zinc finger C2H2 domain of egr3. Once again, sgRNAs efficiency was tested by High Resolution Melt Analysis (HRMA) following by co-injection of the individual guides with Cas9 mRNA into the single cell of a developing zebrafish embryo (Fig. 5C). Targeted knockout of *egr3* resulted in a statistically significant decrease in transcript levels of *il1β* (*p < *0*.*05*) (Fig. 5D), suggesting in important role for egr3 in transcription of *il1b*. Interestingly, knockout of *junbb*, the zebrafish ortholog of the AP-1 complex member JUNB, showed a downward tendency following targeted knockout as previously described (see Supplementary Fig. S5). However, the results were not consistent and the effect was not statistically significant. We hypothesised that this was probably due to alternate roles for junbb in response to *M*. *marinum*, potentially in other cells types, as well as a possible compensation by other jun regulators such as junba, which we also found enriched in neutrophils (Figs 3B and 4A).

## Discussion

Investigations focused on the early host response to mycobacteria are particularly important, since both epidemiological and genetic studies suggest that the early phases of infection, before active or latent tuberculosis is established, might represent a therapeutic window where the mycobacterium can be eliminated^21^. However the sequence of cellular and molecular events taking place in these early stages of infection have not been elucidated. The establishment of latent or active mycobacterial infection is dependent on the pathogen’s ability to withstand the bactericidal properties of macrophages and neutrophils during the initial infection. Although somewhat contradictory reports err on the side a protective role early in infection, the mechanisms by which neutrophils exert antimicrobial properties against mycobacteria remain unclear. One such study found that neutrophils do not phagocytise extracellular *M*. *marinum*, rather that neutrophils were present in the granuloma at 2–4 dpi where they exerted a host-protective role through efferocytosis of infected macrophages and killing of internalised bacteria through NADPH oxidase-dependent mechanisms^5^. This is in line with our findings where we see neutrophils recruited to sites of initial infection, but do not detect them phagocytising bacteria.

The model created in this study provides a powerful tool to obtain the active transcriptome of neutrophils, allowing for the first time to perform an in depth, robust comparison of neutrophils in acute *M*. *marinum* infection versus normal tissues. Our analysis revealed an upregulation of several structural and effector components of the inflammasome pathway in zebrafish in response to *M*. *marinum* infection, including *caspase b*, *wu:fb15h11* (*NLR3C* orthologue) and *il1β*. Using genome engineering to knockout several of upregulated candidate genes, we show for the first time that neutrophils provide an early source of il1β, using an inflammasome-dependent mechanism, in response to *M*. *marinum* infection. It is largely accepted that monoctyes and dendritic cells are the primary source of IL-1β in the lungs of tuberculosis-infected mice^22^. However, the source and role of IL-1β in the early phase of infection before granuloma formation has not been documented. Although previous studies have indicated that *M*. *marinum*, *M*. *tuberculosis* and *M*. *Kansasii* can activate inflammasome pathways in macrophages^23–25^, here we show for the first time that early response of neutrophils to *M*. *marinum* involves inflammasome activation.

Genome-wide analyses of this nature provide a qualitative method to investigate multiple layers of transcriptional complexity simultaneously. Here we report active upregulation of key components of the inflammasome complex in responding neutrophils. As described above, we see no *M*. *marinum* phagocytosis by neutrophils at the time point at which the transcriptional profiling was performed (3 dpi). We thus presumed that inflammasome activation at this stage of infection likely occurs independently of phagocytosis. This is in line with findings that other ‘danger’ signals active during innate immune phase, including pathogen-associated molecular patterns recognized by cell surface receptors, could activate the inflammasome^26^. We also detect an enrichment of number of factors previously recognised to be involved in inflammasome activation in mammals. Those findings together provide us with a hint as to the mechanisms governing the inflammasome function in neutrophils in *M*. *marinum* infection. Those include thioredoxin interacting protein (TXNIP), a previously recognised NLRP3 binding partner, which upon ROS-dependent activation of the inflammasome dissociates from thioredoxin and binds NLRP3. TXNIP deficiency in mice impairs the activation of the NLRP3 inflammasome and subsequent secretion of IL1β^27^. Furthermore, we detect upregulation of leukotriene B4 receptor 2b (*ltb4r2b*), in line with previous reports showing that LTB4 production is triggered upon inflammasome activation^28, 29^. Our data also reveals a putative role for Ca^2+^ influx. Purigenic receptors for extracellular nucleotides such as ATP, which is abundant at sites of inflammation^30^, include the P2X subfamily of receptors which are ligand-gated Ca^2+^-permeable channels that mediate Ca^2+^ influx^30, 31^. P2X_7_R activation has been shown to cause a sustained intracellular increase in Ca^2+^, loss of K+, NLRP3 inflammasome activation and subsequent il1β secretion in neutrophils^31^. Our data suggest a similar role in inflammasome activation for purinergic receptor P2X (*p2rx2*), upregulated in neutrophil nuclei upon *M. marinum* infection. Interestingly, IL-1β expression has been reported to induce expression of matrix metalloproteinase 9 (*MMP9*)^32, 33^. MMP9 is highly expressed in human tuberculosis, in mouse models of tuberculosis and in *M*. *marinum* infection. It has previously been shown that following *M*. *marinum* infection in zebrafish *mmp9* is induced in epithelial cells resulting in enhanced macrophage recruitment and increase bacterial growth^3^. However, the same study showed that mmp9 was largely expressed by distal neutrophils, rather than at the site of infection. However, our analysis, performed on neutrophils at the sites of infection, clearly shows enrichment and active nuclear transcription of *mmp9*.

The mechanisms by which IL-1β-driven inflammation augments host resistance against mycobacterial infection have not yet been fully elucidated. A recent investigation demonstrated that IL-1 confers resistance to mycobacteria through the induction of eicosanoids and in particular PGE2 synthesis. In turn, PGE2 induction limits type I interferon (IFN) production, known to promote bacterial virulence and disease exacerbation, ultimately containing bacilli replication^22^. Furthermore, IL-1β from neutrophils may act directly on infected macrophages. Treatment of *M*. *tuberculosis*-infected macrophages with recombinant IL1β results in upregulation of TNF and TNFR1 and the subsequent activation of caspase-3. In turn, the activation of caspase-3 results in the apoptosis of M. tuberculosis-infected macrophages and subsequent efferocytosis of infected apoptotic macrophages leading to control of bacterial replication^34^. Early studies showed the importance of IL1β in granuloma formation, whereby coated beads were capable of inducing large granulomas in lung tissue. IL-1 type 1 receptor-deficient (*IL-1R* ^−/−^) mice, where IL-1 is unable to exert an effect, showed higher susceptibility to tuberculosis, impaired granuloma formation containing fewer macrophages and lymphocytes but abundant granulocytes^35^. We have shown that knockout of *caspase b*, presumably resulting in a decrease in il1β secretion results in less macrophages being recruited to sites of infection, but does not affect the early recruitment of neutrophils. Therefore neutrophil-derived IL-1β may contribute to the containment of the disease through the recruitment of macrophages to sites of infection and the subsequent granuloma formation.

Expression of IL1β is tightly controlled by both the transcriptional regulation of pro-IL1β and a second signal that results in proteolytic processing of the protein to the mature 17 kDa form. Regulatory network analysis of community-generated transcription factor binding sites combined with *de novo* analysis led us to predict a role for egr3 in regulating the transcription and thus priming of *il1β*. egr3 has recently been shown to regulate the expression of ~330 genes, 35% of which are involved in immune responses and inflammatory processes, and interestingly exhibits 15% crosstalk with the NF-*κ*B signalling^36^, a well characterised inflammasome priming pathway in mammalian macrophages. In addition to binding directly to the regulatory elements of *il1β*, egr3 may affect the transcription of *il1β* indirectly by binding other transcription factors. This is particularly important since transcriptional modules encode functional programmes of the inflammatory response. Gaining an understanding of the organisation of these transcriptional programmes provides an opportunity to manipulate the immune response.

In summary, we present a powerful new model to study the regulatory programming of neutrophils using *in vivo* biotagging to capture the active nuclear transcriptome. This model allows us for the first time to uncover a novel role for neutrophils in the initial stages of *Mycobacterium marinum* infection. We show that neutrophils serve as an early source of il1β that may contribute to the containment of the disease by recruiting macrophages to sites of infection and hence enabling granuloma formation. We provide insight into two modes of regulatory control of il1β production in neutrophils – at the transcriptional level by *egr3* and by inflammasome-mediated processing of il1β at the post-translational level.

## Materials and Methods

### Zebrafish maintenance and strains

This study was carried out in compliance with local ethical approval from the University of Oxford and using procedures authorized by the UK Home Office in accordance with UK law (Animals Scientific Procedures Act 1986). Zebrafish were maintained as described^37^. Wild-type embryos for transgenesis were obtained from AB or AB/TL mix strains.

### Transgenic fish line generation and maintenance

The *TgBAC*(*mpx:BirA-2A-citrine*)^*ox122*^ transgenic line was generated by BAC recombination as previously described^22^. Briefly, pGEM BirA-2A-Citrine-SV40pA-FRT-Kan-FRT donor plasmid for BAC transgenesis was cloned by fusion PCR of HA-BirA-2A amplified from PMT-HA-BirA-2A-mCherryRas^11^ and insertion into pGEM-GFP-SV40pA-FRT-Kan-FRT where the GFP ORF was been replaced with Citrine. The pGEM BirA-2A-Citrine-SV40pA-FRT-Kan-FRT donor plasmid is available from Addgene. Subsequently, the BirA-2A-Citrine-sv40-FRT-kan-FRT cassette was recombined into the first exon of a BAC clone containing the neutrophil-specific myeloperoxidase gene (*mpx*). In a second recombination step, an iTol2-Ampicillin cassette (provided by Prof Kawakami) was introduced into the BAC backbone as previously^38, 39^. Wildtype embryos were injected at the one-cell stage with 200 ng/μL purified BAC DNA and 100 ng/μL tol2 transposase mRNA. Putative founders were identified by citrine expression in neutrophils by outcrossing to wildtype fish. *Tg*(*mpeg1:EGFP*)^*gl22*^ were used in the macrophage enumeration experiments^40^.

### *M*. *marinum* culture preparation

*M*. *marinum* (ATCC # BAA-535) was grown at 30 °C in Middlebrook 7H9 broth supplemented with 0.5% BSA, 0.2% glucose, 0.2% glycerol, 0.085% NaCl, catalase and 0.05% Tween 80. Cultures were grown to an optical density at 600 nm and maintained at −80 °C in freezing medium (30% glycerol, 0.05% Tween 80). *M*. *marinum* glycerol stock was transformed by electroporation (2.5 kV, 4.5 ms pulse) with a pTEC27 expression vector containing a tdTomato reporter (Addgene, cat # 30182) and plated on 7H10 agar, supplemented with 100 μg/mL hygromycin. Single cell suspension from transformed colonies were prepared as previously described^8^.

### *M*. *marinum* injections

Embryos were injected at the 32–512-cell stage (20 CFU *M*. *marinum* in PBS, 0.1% phenol red). Embryos were reared under standard conditions and E3 replaced daily.

### Whole-mount *in situ* hybridisations

Whole-mount *in situ* hybridisations were performed as previously described with minor modifications^41^. The templates for antisense *in situ* probes were generated from PCR amplified templates using gene-specific primers and cDNA from  wildtype zebrafish embryos (3 dpf). The full primer list is available in Supplementary Information. 1 μg of template DNA was transcribed using T7 RNA polymerase (Promega, #P207B) in the presence of digoxygenin (DIG)-labelled dNTPs (Roche, #12430721). *In situ* probes were purified by two passes through G-50 micro-columns (GE Healthcare, #28-9034-08). Embryos were fixed in 4% paraformaldehyde (PFA) at 4 °C, dehydrated and stored in methanol MeOH. Embryos were rehydrated in an incremental series of MeOH/phosphate-buffered saline (PBS) with 0.1% Tween (PBST) washes followed by 5 minute in PBST.

Embryos were permeabilised with proteinase K in PBST/diethylpyrocarbonate (DEPC) (10 µg/mL) for 30 minutes, followed by glycine solution in PBST/DEPC (2 mg/mL) for 10 minutes and then post-fixed with paraformaldehyde (PFA) (20 minutes at RT). Embryos were equilibrated with hybridization buffer (50% Formamide, 1.3× SSC pH 5.0, 5 mM EDTA pH 8.0, 200 μg/mL  Baker’s yeast tRNA, 0.2% Tween-20, 0.5% 3-[(3-cholamidopropyl)dimethylammonio]-1-propanesulfonate (CHAPS), 100 μg/mL Heparin) and incubated overnight with DIG-labeled probes at 68 °C). Embryos were blocked with 20% sheep serum + 2% Boehringer Blocking Reagent (Roche, cat # 11096176001). Probes were detected with an anti-DIG-alkaline phosphatase antibody (Roche, cat # 11093274910) and the signal was developed using BM Purple AP (Roche, cat # 11442074001).

### Whole-mount immunofluorescence and imaging

Embryos/larvae were fixed in 4% PFA in Phosphate buffered saline (PBS) (0.1 M, pH 7.4) for 20 minutes at RT. Embryos were then rinsed 3 × 15 minutes in PBT (2% DMSO, 0.5% Triton in PBS), incubated in block (10% donkey serum in PBT) for 2 hours at RT, followed by incubation with primary antibody (1:200 in block) O/N at 4 °C. Embryos/larvae were then washed 3–5 × 1 hour at RT + O/N at 4 °C in PBT and then incubated with secondary antibody (1:500 in PBT) followed by a final wash step of 6–8 × 1 hour at RT + O/N at 4 °C in PBT. Primary antibodies used in this study were, mpx (GeneTex, cat # GTX128379). The secondary antibody used was AlexaFluor-488 IgG (Invitrogen, cat # A-11008).

### Microscopy

Images of neutrophil and *M*. *marinum* were imaged on an Olympus MVX10 stereomicroscope with a 6.3× objective. Images for granuloma quantification and macrophage enumeration were taken on a Zeiss 780 confocal microscope with 40× and 20× objectives respectively.

### ‘Biotagged’ nuclei isolation

For nuclei isolation, zebrafish embryos were anaesthetised with 0.01% Tricaine 0.01% and aggregates of mycobacterium dissected based on tdTomato fluorescence. Embryos were washed in hypotonic buffer H (20 mM HEPES (pH 7.9), 15 mM MgCl_2_, 10 mM KCl, 1 mM DTT, and 1 X Complete protease inhibitor) and subsequently re-suspended in 500 μL of buffer H. Embryos were transferred to a Dounce homogenizer and dissociated by 10 strokes with the loose fitting pestle A and incubated on ice for 5 minutes. Further dissociation was carried out by 10 strokes with tight fitting pestle B, every 5 minutes for 15 minutes. Nuclei were collected by centrifugation (2000 g, 4 °C) and re-suspended in 1 mL buffer NDP (10 mM HEPES (pH 7.9), 40 mM NaCl, 90 mM KCl, 0.5 mM EDTA, 0.5 mM spermidine, 0.15 mM spermine, 1 mM dithiothreitol and 1 X Complete protease inhibitor). For nuclei purification, nuclei were incubated with 5 mg of M-280 streptavidin-coated dynabeads with rotation for 30 minutes at 4 °C. A flow-based system based on the previously published protocol by was used to capture the nuclei bound on the streptavidin beads. A 10 mL seriological pipette (VWR, cat # 8913–898) attached to a 1 mL micropipette tip (Rainin reach pipet tip, cat # RT-L1000S), both pre-treated with NPB/BSA for 30 minutes, and added to a MiniMACS separator magnet (OctoMACS Separator, Miltenyl Biotec, cat # 130–042–109). A two-way stopcock (Biorad cat # 732–8102) was connected to the end of the 1 mL micropipette tip via a piece of Tygon tubing (Fisher Scientific, cat # 8220) and the flow-rate set to ~0.75 mL/min. The nuclei beads suspension was diluted by addition of 9 mLs of nuclei pulldown buffer (NPB) with 0.01% Triton X-100 (NPBt) and added to the slow-flow setup. The tip was subsequently removed from the stand and the nuclei-beads released from the tip by slowly pipetting 1 mL of NPBt in and out of the tip. The solution was then diluted again to 10 mL with NPBt and added again to the slow-flow setup. Nuclei-beads were eluted in 1 mL of NPBt as described above and the NPBt removed using a magnetic stand (DynaMag^TM^-2 magnet, Invitrogen, cat # 12321D). Nuclei-beads were then processed for RNA extraction.

### RNA Extraction, library preparation and RNA-seq analysis

RNA extraction and DNAse treatment were carried out using the RNAqueous^®^-Micro Kit (Life Technologies, cat # AM1931). RNA integrity was checked with a RNA pico chip (Agilent Technologies, cat # 5067–1513) using the Agilent 2100 Bioanalyzer.

cDNA was synthesized and amplified from 100 pg–1 ng of input RNA using SMART-seq^TM^ v4 ultra low input kit for RNA (Clontech laboratories, cat #’s 634888, 634889, 634890, 634891, 634892, 634893, and 634894). Sequencing libraries were prepared using the Nextera XT DNA library preparation kit and sequencing performed on a NextSeq 500 platform using a NextSeq™ 500 High Output Kit for the generation of 80 base pair paired end reads (150 cycles) (Illumina, cat # FC-404-1002).

### RNA-seq analysis

Read quality was evaluated using FastQC^42^. Adapter contamination was removed using Scythe^43^ and reads further trimmed based on base quality using Sickle^44^. Adapter-removed and quality trimmed reads were mapped to the (Jul. 2014 Zv10/danRer10 assembly) version of the zebrafish genome using STAR (v.2.4.2a) splice-aware aligner^45^. Count tables were generated using subread FeatureCounts (v1.4.5-p1q), with standard parameters^46^.

Differential expression was carried out using DESeq2 R package^47^. Functional classification using the Panther system (http://www.pantherdb.org/) with gene ontology (GO) were carried out on genes that were found to be differentially expressed with an adjusted p-value of <0.05^48, 49^. Statistical overrepresentation was calculated using hypergeometric and Fisher’s exact test. Data generated in this study were submitted to GEO (GSE92740).

Promoter motif analysis was performed using Homer^50^. Motifs were obtained from JASPAR^51^. Egr3 and Egr4 sites were obtained from CIS-BP^52^. Promoters were defined as −500 to +1000 of TSS using Refseq annotation obtained from UCSC genome browser for danRer7.

### sgRNA and Cas9 mRNA synthesis

sgRNA template DNA was generated with a unique oligonucleotide encoding the T7 RNA polymerase recognition site, the sgRNA target sequence and an overlap with the tracrRNA. The full list of gene-specific oligonucleotides, synthesized by IDT (Leuven, Belgium) used in this protocol is available in Supplementary Information. Each gene-specific sgRNA oligo was first annealed with the universal reverse oligo containing tracrRNA sequence (AAAAGCACCGACTCGGTGCCACTTTTTCAAGTTGATAACGGACTAGCCTTATTTTAACTTGCTATTTCTAGCTCTAAAAC) and subsequently amplified by PCR. *In vitro* transcription was performed with 500–1000  ng purified DNA template for using the T7 RNA polymerase kit (NEB) for 4 hours at 37 °C, and sgRNA purified using the Megaclear kit (Ambion, cat #AM1908). Cas9 mRNA was *in vitro* transcribed from plasmid MLM3613 (Addgene, cat #42251) using the mMESSAGE mMACHINE T7 kit (Ambion, cat #AM1344).

### HRMA analysis

Genomic DNA was extracted from single embryos at 24 hpf using the Purelink Genomic DNA minikit (Invitrogen). Primers were designed to generate a ~200 bp product spanning the cut site. Hotshot Diamond PCR mastermix (Client Lifescience, #HS002-TS) was used to perform PCR with LC Green Plus dye (BioFire Diagnostics, #BCHM-ASY-0005). Reaction solutions were cycled on a C1000 Touch^TM^ Bio-Rad thermal cycler.

### Quantitative PCR

RNA extraction and cDNA synthesis were carried out using the RNAqueous^®^-Micro Kit (Life Technologies, cat # AM1931) and Superscript III Reverse Transcriptase (Invitrogen, #18080093), respectively. Quantitative PCR was performed using Fast SYBR Green Master Mix (Applied Biosystems, #4385612) on a 7500 Fast Real-Time PCR system (Applied Biosystems). Gene-specific primers used for il1β were 5′-GGACTGTTCAGATCCGCTTG and 3′-TCATTTTGTGCTGCGAAGTC. Expression levels were normalised to β-actin.

### Statistical analysis

Graphs and statistical analyses were generated by Graphpad prism, version 6. Not significant, p > 0.05; *p < 0.05; **p < 0.01; ***p < 0.001; ****p < 0.0001. Briefly, statistical significance for quantification of neutrophil presence (2, 3, 4,5 days post infection) was performed by one-way ANOVA. Statistical significance of changes in bacterial burden as measured by *M*. *marinum* fluorescence was determined by a two-tailed unpaired students t-test with Welch’s correction. Statistical significance of larvae with cording phenotype was determined by chi-squared test. Statistical significance of percentage of larvae where macrophages phagocytise *M*. *marinum* was measured by Fisher’s exact test. Statistical significance of il1β mRNA levels was determined by a two-tailed unpaired students t-test with Welch’s correction.

## Electronic supplementary material

Supplementary Information

Dataset 1
