# Supplementary material for: Active nuclear transcriptome analysis reveals inflammasome-dependent mechanism for early neutrophil response to Mycobacterium marinum
Source: Sci Rep. 2017 Jul 26;7:6505. doi: 10.1038/s41598-017-06099-x (PMC5529371; doi:10.1038/s41598-017-06099-x)

Active nuclear transcriptome analysis reveals inflammasome-dependent mechanism for early neutrophil response to *Mycobacterium marinum*

Amy Kenyon[1,2], Daria Gavriouchkina[1], Jernej Zorman[1], Giorgio Napolitani[2], Vincenzo Cerundolo[2] and Tatjana Sauka-Spengler[1, §]

Supporting Information

Figure S1: Generation of BirA driver line

A. Schematic of the mpo BAC donor construct, containing HA-tagged BirA (orange), a ribosomal skipping motif - 2A (grey), citrine reporter (green), polyA tail (white), followed by FRT recombination sites (turquoise) flanking a kanamycin selection cassette. Ampicillin selection cassette, not amplified as a part of recombination cassette, is used as a selection marker for *E. coli* during plasmid DNA isolation. Red arrows indicate position of primers used for amplification and recombination into the mpo BAC. **B.** Schematic of mpo modified BAC DNA with HA-tagged BirA (orange), a ribosomal skipping motif - 2A (grey), citrine reporter (green), polyA tail (white), followed by the remaining FRT sites (turquoise) recombined into the first exon, with a BAC-specific ampicillin-Tol2 cassette (iTol2) in pink and yellow.

Figure S2: Technical reproducibility of replicates

A. Scatterplot of log2 fold differences between biological duplicates for representative neutrophil nuclear samples –*Mm* vs –*Mm* (i), +*Mm* vs +*Mm* (ii) and +*Mm vs* –*Mm* (iii). **B.** Table presenting Pearson correlation coefficients to all possible pairwise comparisons of replicates/samples.

Figure S3: sgRNA-mediated gene knockdown of wu:fb15h11

A. *Wu:fb15h11* sgRNA tests for efficiency in inducing DNA double-strand breaks. SgRNAs were injected into the 1-cell stage and genomic DNA extracted at 24 hpf. High resolution melting curves from 3-4 individual embryos injected either with Cas9 mRNA only (green) or co-injected with Cas9 mRNA and *wu:fb15h11* sgRNA (red) are shown.

Figure S4: Enumeration of neutrophil numbers following caspase b knockdown

A. Bar graph representing neutrophil numbers counted on days 2 - 5, following injection of *M. marinum* at the 32-512 cell stage into control (injected with Cas9 mRNA only) or caspase b knockout embryos (co-injected with caspase b sgRNAs and Cas9 mRNA).

Figure S5: sgRNA-mediated gene knockdown of junbb

A. *junbb* sgRNA tests for efficiency in inducing DNA double-strand breaks. High resolution melting curves from 3-4 individual embryos injected either with cas9 mRNA only (green) or co-injected with Cas9 mRNA and *junbb* sgRNA (red) are shown. **B.** Bar graph representing il1β mRNA levels measured at 3 dpi by qPCR. Statistical significance was determined by two-tailed unpaired students t-test with Welch's correction. Wildtype fish were co-injected with cas9 mRNA and 3 sgRNAs for targeted knockout of junbb or Cas9 mRNA only at the one cell stage, followed by injection of *M. marinum* at the 32-512 cell stage.

Supplemental data - Primers

**Primers for in situ probes synthesis**	**Sequence**
5' junbb sp6 sense	GATTTAGGTGACACTATAGAAAATGGAGCAGCCGTTTTA
3' junbb t7 antisense	TAATACGACTCACTATAGGGAAACGCCTCCATCTTACTGG
5' hbegfa sp6 sense	GATTTAGGTGACACTATAGGAGTCTCGGAGGGATTACTTTT
3' hbegfa t7 antisense	TAATACGACTCACTATAGGGGCTCTCCTGCGAACTTCTTT
5' il1b sp6 sense	GATTTAGGTGACACTATAGGCAGAGGAACTTAACCAGCTC
3' il1b t7 antisense	TAATACGACTCACTATAGGG CGCACTTTATCCTGCAGCTC

5' il8L1 sp6 sense	GATTTAGGTGACACTATAGCTCCACACACACCGGCTTC
3' il8L1 T7 antisense	TAATACGACTCACTATAGGGTTTTATTAATCATACAAGCAGATGTCA
5' egr3 sp6 sense	GATTTAGGTGACACTATAGGCGGACAAGATCCCTATTACC
3' egr3 T7 antisense	TAATACGACTCACTATAGGGAGATGGTCACTGCGACTGAA
5' egr4 sp6 sense	GATTTAGGTGACACTATAGCTGAACACCGTGGACTTCAG
3' egr4 T7 antisense	TAATACGACTCACTATAGGGCGCGCTGTTTGAGGTGAAC
5' ier2 sp6 sense	GATTTAGGTGACACTATAGTGAATGAAACCACGGTGAAAGT
3' ier2 T7 antisense	TAATACGACTCACTATAGGGTGTGGATTCTTCAGCAAGTGA
5' tnfb sp6 sense	GATTTAGGTGACACTATAGGCAGCATGGTGAGATACGAA
3' tnfb t7 antisense	TAATACGACTCACTATAGGGCGTCACATTAGCTTGCGATAA
5' cepbp sp6 sense	GATTTAGGTGACACTATAGGTATGCAAGCAGCCAGTCAA
3' cepbp t7 antisense	TAATACGACTCACTATAGGGCAATCGACGGCAGAAAACTT
5' fosl1a sp6 sense	GATTTAGGTGACACTATAGAGAAAGCAGCTCTGGATCGT
3' fosl1a t7 antisense	TAATACGACTCACTATAGGGGGGTGAGGATTGTGGGTGAA
5' junba sp6 sense	GATTTAGGTGACACTATAGACTCGTTTCTTTCTGCTTATGGT
3' junba t7 antisense	TAATACGACTCACTATAGGGAAACGACTTGATCTTGGGCG
5' caspase b sp6 sense	GATTTAGGTGACACTATAGTGATGTGCTCGAGGATCTTG
3' caspase b t7 antisense	TAATACGACTCACTATAGGGCCCTCGATTGGATTCCTGTA

5' wu:fb15h11 sp6 sense	GATTTAGGTGACACTATAGAGCTGGAGACGACAATGAAGA
3' wu:fb15h11 t7 antisense	TAATACGACTCACTATAGGGTGAGGGAAAAAGAGCTGAAGA

**Primers for sgRNA synthesis**	**Sequence**
caspase b 1	**GAAATTAATACGACTCACTATAGGG** TGACAATAAAGATGCCATTGT GTTTTA GAGCTAGAAATAGC
caspase b 2	**GAAATTAATACGACTCACTATAGG** GGAGTGGCTGCTGAGATCTCGTTTTA GAGCTAGAAATAGC
caspase b 3	**GAAATTAATACGACTCACTATAGG** GTTTCGGTTGTGTTGTGTAT GTTTTA GAGCTAGAAATAGC
wu:fb15h11 1	**GAAATTAATACGACTCACTATAGG** GCTGAAGAGAAGGAGAATC GTTTTA GAGCTAGAAATAGC
wu:fb15h11 2	**GAAATTAATACGACTCACTATAGG** GCTTCCCTTAAATTTCAGA GTTTTA GAGCTAGAAATAGC
wu:fb15h11 3	**GAAATTAATACGACTCACTATAGG** GGGACGAGATCTGCTGCTGC GTTTTA GAGCTAGAAATAGC
junbb 1	**GAAATTAATACGACTCACTATAGG** GCATCAAGGCGGAACGCAAGGTTTTAGAGCTAGAAATAGC
junbb 2	**GAAATTAATACGACTCACTATAGG** GCAAACTAGAACGCATCGCGGTTTTA GAGCTAGAAATAGC
junbb 3	**GAAATTAATACGACTCACTATAGGG**AACTCAAGCAGAAGGTCCTGGTTTTA GAGCTAGAAATAGC

egr3 1	**GAAATTAATACGACTCACTATAGG**GTCCGGCCGAGAACTGTGACGTTTTA GAGCTAGAAATAGC
egr3 2	**GAAATTAATACGACTCACTATAGG**GCGATCCGATGAGTTGACGGTTTTA GAGCTAGAAATAGC
egr3 3	**GAAATTAATACGACTCACTATAGGG**TCCAGTGCCGCATCTGCATGGTTTTA GAGCTAGAAATAGC

Active nuclear transcriptome analysis reveals inflammasome-dependent mechanism for early neutrophil response to Mycobacterium marinum

Supplementary Figure 1

A i)

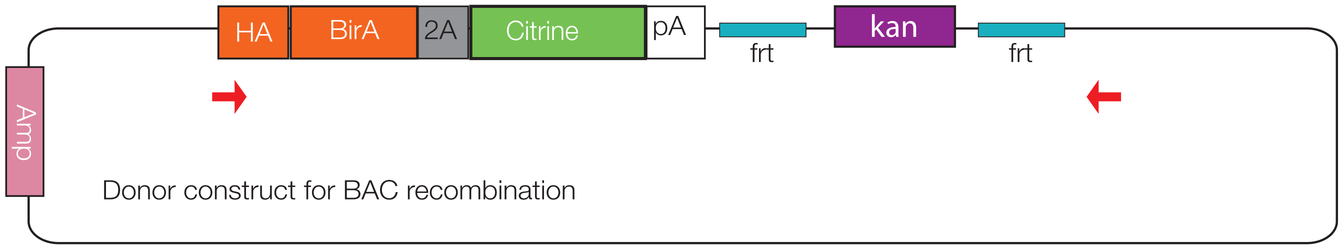

ii)

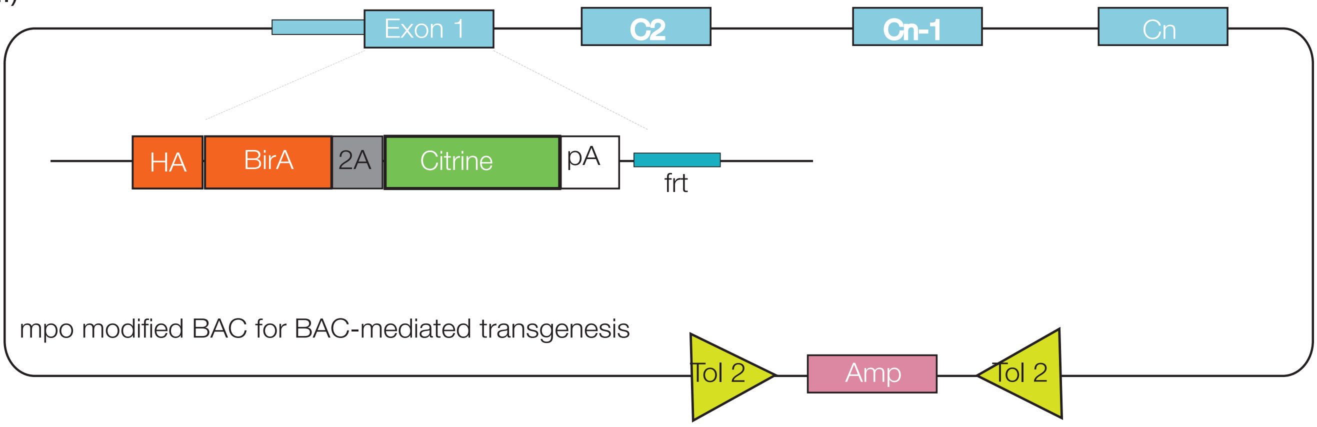

Supplementary Figure 2

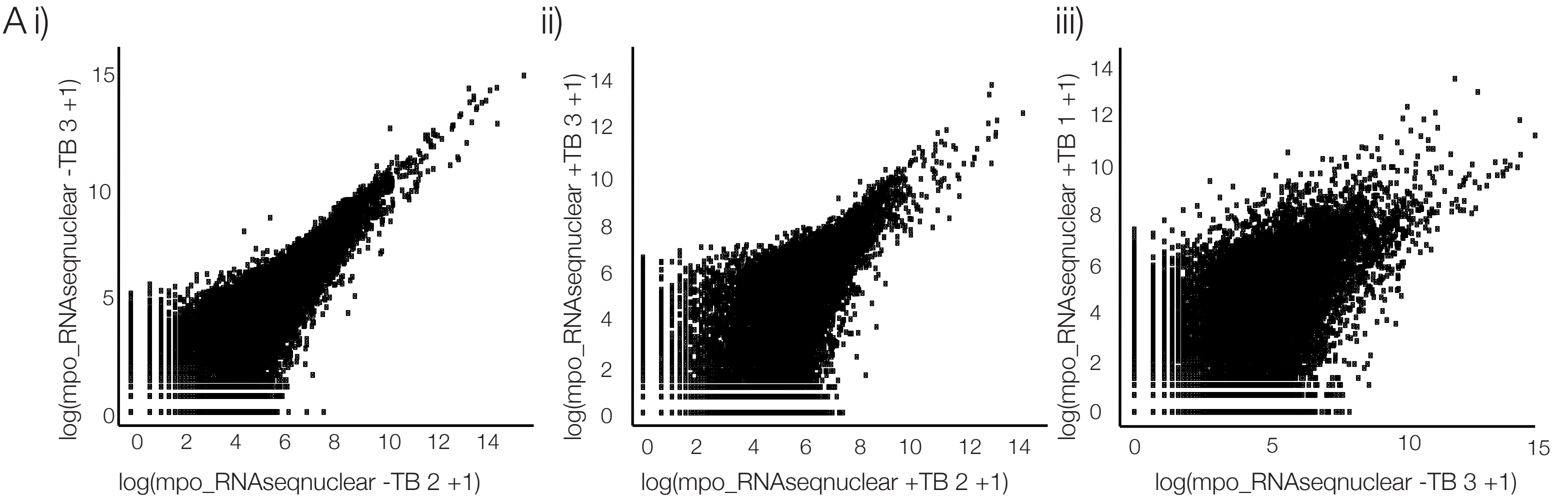

B

Pearson	mpo + TB 1	mpo + TB 2	mpo + TB 3	mpo - TB 1	mpo - TB 2	mpo - TB 3
mpo + TB 1	1	0.8675646	0.8342894	0.8298012	0.8187772	0.7828011
mpo + TB 2	-	1	0.8922661	0.8439331	0.8866421	0.8408514
mpo + TB 3	-	-	1	0.776431	0.765863	0.7593172
mpo - TB 1	-	-	-	1	0.8292914	0.7776135
mpo - TB 2	-	-	-	-	1	0.7731537
mpo - TB 3	-	-	-	-	-	1

Supplementary Figure 3

A

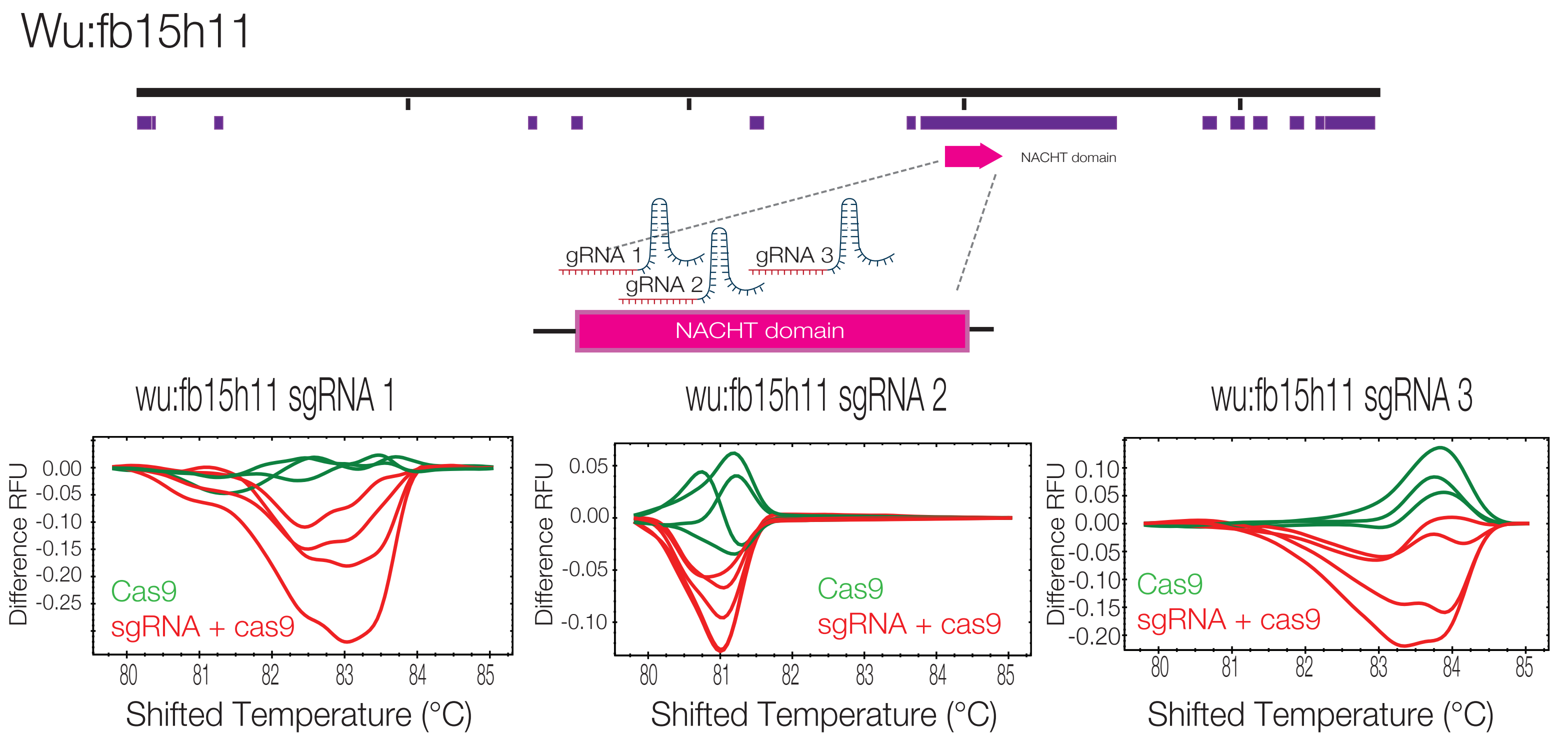

Supplementary Figure 4

A

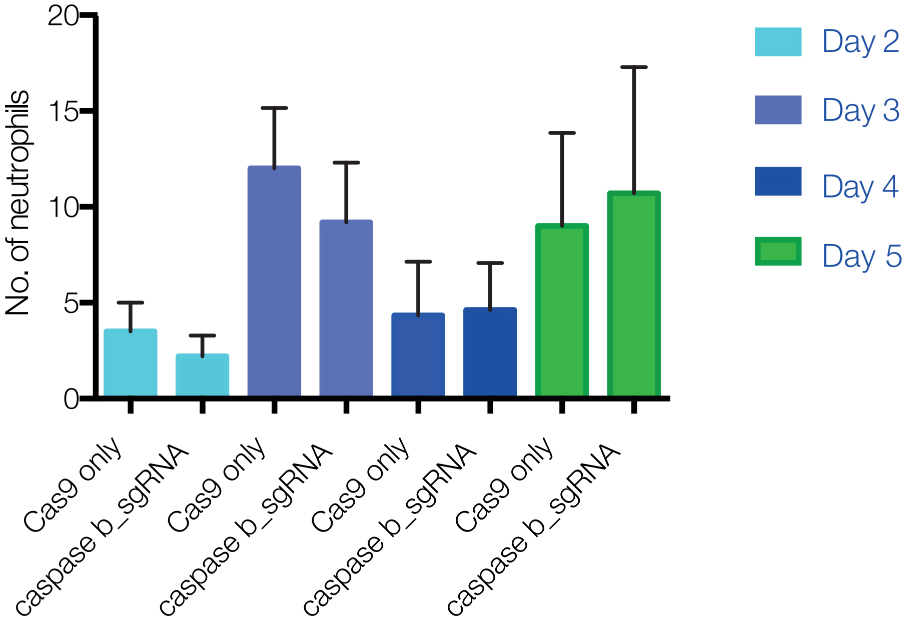

Supplementary Figure 5

A

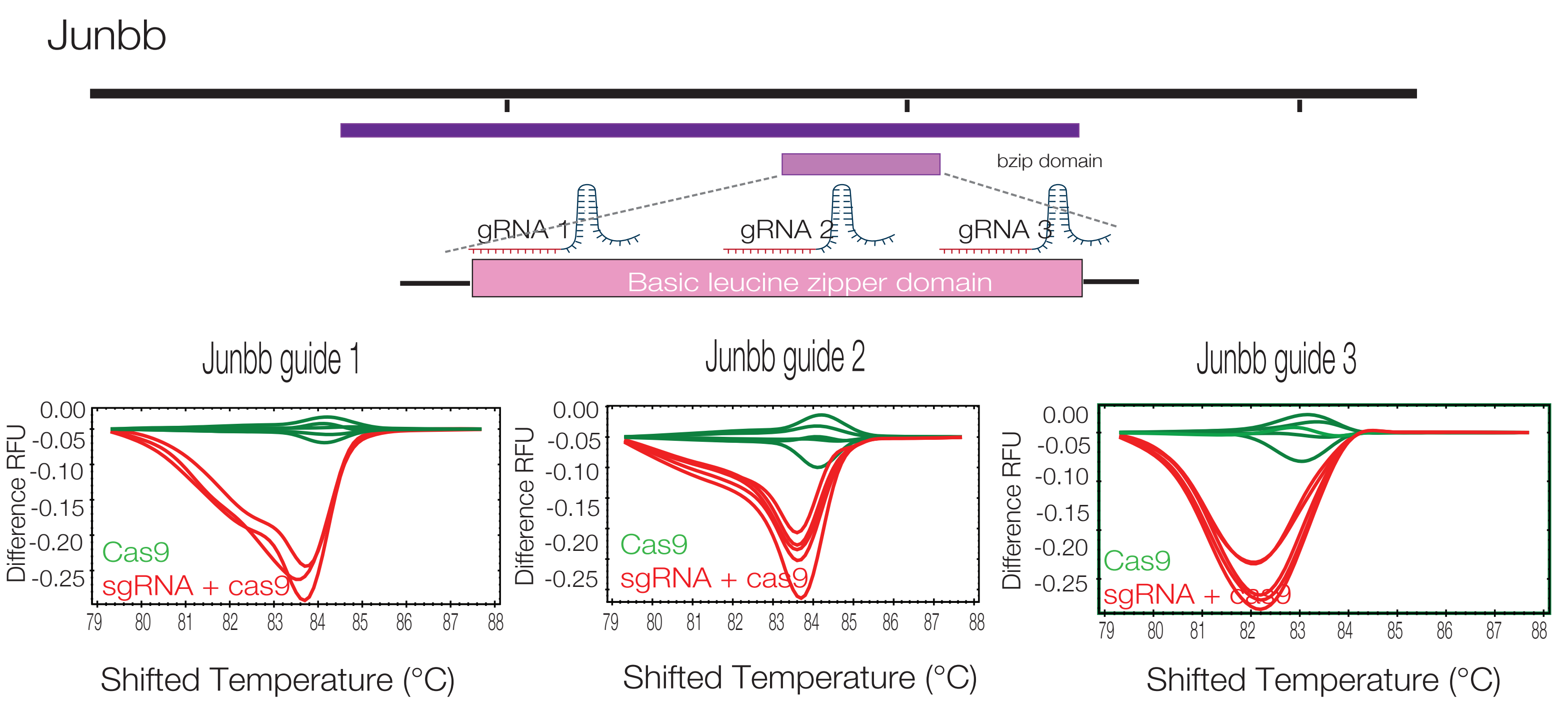

B

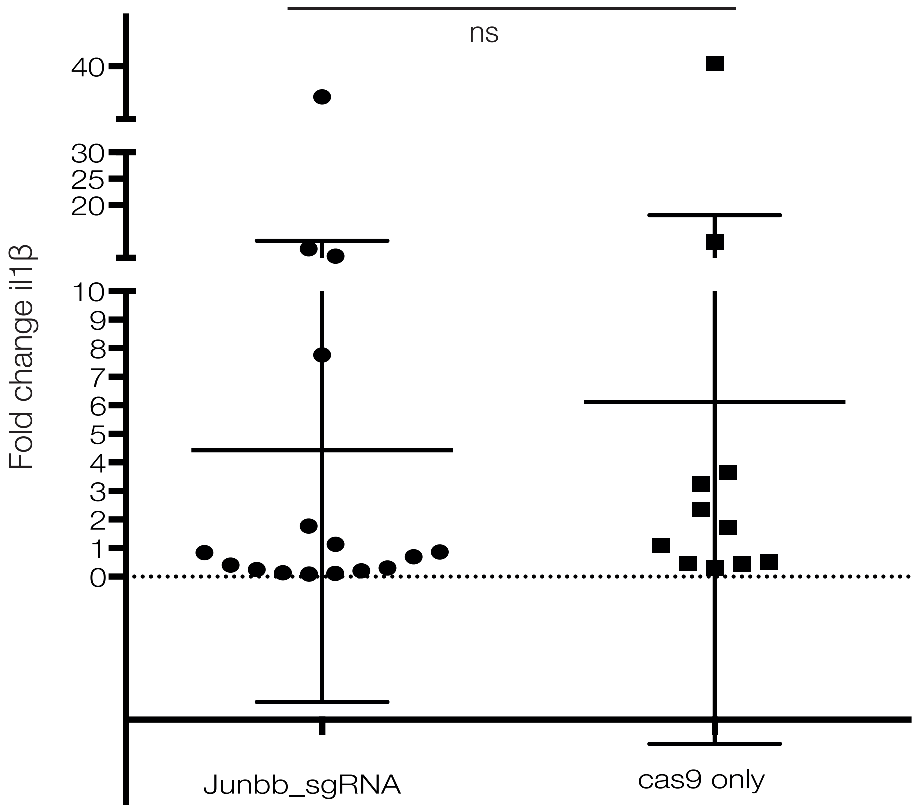

Supplement: Supplementary file 1 — Supplementary Information [file 41598_2017_6099_MOESM1_ESM.pdf]
